# Synergistic Antimicrobial Activity Between the Broad Spectrum Bacteriocin Garvicin KS and Nisin, Farnesol and Polymyxin B Against Gram-Positive and Gram-Negative Bacteria

**DOI:** 10.1007/s00284-017-1375-y

**Published:** 2017-10-20

**Authors:** Hai Chi, Helge Holo

**Affiliations:** Faculty of Chemistry, Biotechnology and Food Science, Norwegian University of Life Sciences, POB 5003, 1432 Ås, Norway

**Keywords:** Synergy effect, Bacteriocin, Garvicin KS, Antimicrobials, Pathogens

## Abstract

The increasing emergence of antibiotics resistance is of global concern. Finding novel antimicrobial agents and strategies based on synergistic combinations are essential to combat resistant bacteria. We evaluated the activity of garvicin KS, a new bacteriocin produced by *Lactococcus garvieae*. The bacteriocin has a broad inhibitory spectrum, inhibiting members of all the 19 species of Gram-positive bacteria tested. Unlike other bacteriocins from Gram-positive bacteria, garvicin KS inhibits *Acinetobacter* but not other Gram-negative bacteria. Garvicin KS was tested in combination with other antimicrobial agents. We demonstrated synergy with polymyxin B against *Acinetobacter* spp. and *Escherichia coli*, but not against *Pseudomonas aeruginosa*. Similar effects were seen with mixtures of nisin and polymyxin B. The synergistic mixtures of all three components caused rapid killing and full eradication of *Acinetobacter* spp. and *E. coli*. In addition, garvicin KS and nisin also acted synergistically against *Staphylococcus aureus*, indicating different in modes of action between the two bacteriocins. Both bacteriocins showed synergy with farnesol, and the combination of low concentrations of garvicin KS, nisin and farnesol caused rapid eradication of all the *S. aureus* strains tested. Its broad inhibitory spectrum, rapid killing, and synergy with other antimicrobials makes garvicin KS a promising antimicrobial.

## Introduction

Infections caused by antibiotics resistant pathogens from both Gram-positive and Gram-negative bacteria have become of global concern. In addition, the slow discovery and development of new antibiotics is unable to catch up the rapid increasing of antibiotics resistance [5, 24]. Searching for alternative antimicrobials for inhibition and elimination of the antibiotics resistant pathogens as well as reducing resistance evolution is essential. Antimicrobial peptides represent a source of unexplored compounds with a potential to kill antibiotic resistant bacteria [27]. Their modes of action are not fully understood, but are different from the commonly used antibiotics. The antimicrobial peptides produced by bacteria are known as bacteriocins. Bacteriocins usually have narrow inhibitory spectra, but from Gram-positive bacteria several bacteriocins with wide inhibitory spectra are known. The bacteriocins of lactic acid bacteria are of particular interest, since many are being used in foods and their bacteriocins may contribute to enhance shelf life and food safety. Based on primary structure, these bacteriocins have been grouped into class I, lantibiotics containing modified residues, and class II without modified residues [6].

The best-studied of these bacteriocins is the lantibiotic nisin, which inhibits most Gram-positive bacteria but not Gram-negative [12, 30]. However, in the presence of compounds that can destabilize the outer membrane, even Gram-negatives are inhibited [14, 28].

We recently described garvicin KS, a bacteriocin produced by *Lactococcus garvieae* [24]. We report here on its antimicrobial spectrum, which includes Gram-negative bacteria. We show that it can act synergistically with other antimicrobials, including nisin, against several pathogenic bacteria. Moreover, the synergistic mixtures can improve killing kinetics reducing the risk of resistance development.

## Materials and Methods

### Bacterial Strains and Growth Conditions

*Lactococci* were grown in M17 broth (Oxiod, Hampshire, UK) supplemented with 0.4% (w/v) glucose (GM17), *Lactobacilli* and *Pediococci* were grown in deMan, Rogosa and Sharpe (MRS) (Oxoid, Hampshire, UK) medium at 30 °C. *E. coli, Pseudomonas aeruginosa*, and *Acinetobacter* strains were grown in Lysogeny broth (Oxoid, Hampshire, UK) or Mueller Hinton Broth (MHB) (BD Difco, MD, USA) at 37 °C with shaking at 225 rpm. Other strains were grown in Brain Heart Infusion (Oxoid, Hampshire, UK) broth at 30 °C.

### Antimicrobial Agents

Nisin from *Lactococcus lactis* (2.5% purity), polymyxin B sulfate and farnesol were from Sigma-Aldrich, Co. (St. Louis, MO, USA). Crude garvicin KS was prepared by following procedure. 1 L of GM17 broth was inoculated with 1% of an overnight culture of *L. garvieae* KS1546 and incubated at 30 °C for 8 h. The cells were removed by centrifugation at 12,000×*g* for 25 min and 258 g of ammonium sulfate was dissolved in the cell-free supernatant, and the mixture was left at 4 °C for 24 h. The crude bacteriocin was precipitated by centrifugation at 15,000×*g* for 30 min at 4 °C, dissolved in 20 mL water and kept in a boiling water bath for 10 min.

### Inhibition and Checkerboard Assays

Growth inhibition was determined in microtiter plates with 200 µL of growth medium in each well as described by Holo et al. [16]. The minimum inhibitory concentration (MIC_50_) refers to the concentration of antimicrobial agent causing 50% growth inhibition, the MIC_50_ was detected once the OD_600_ of testing strains reaches 0.4–0.5. Bacteriocin unit [2] of garvicin KS was defined as the amount of garvicin KS causing 50% growth inhibition of *L. lactis* IL1403 in this assay. The checkerboard assays using mixture of antimicrobials were performed in microtiter plates as described above.

Interactions between antimicrobial agents were determined by using the fractional inhibition concentration (FIC). The FIC was calculated as follows: FIC = FICa + FICb + FICc, where the FICa means MIC of A in combination/MIC of A alone, FICb means MIC of B in combination/MIC of B, and FICc means MIC of C in combination/MIC of C alone. Effects were considered as synergistic if FIC was ≤0.5 for two components mixture [22] and ≤0.75 for three components mixture [3].

### Time-Kill Assays

Time-kill assays were performed using cultures grown overnight and then diluted 100× in MHB with different concentrations of antimicrobial agents. The assays were done in triplicate. Total viable count (TVC) was determined by plating on Mueller Hinton agar. The agar plates were incubated at 37 °C, and the TVC was estimated after 24 h incubation. Interactions between antimicrobial agents were interpreted as synergistic when they in mixture caused ≥2-log decrease in TVC compared to the antimicrobial agents alone [25].

## Results

### Garvicin KS has a Broad Antimicrobial Inhibition Spectrum

Garvicin KS, produced by *L. garvieae* strain, is composed of three similar peptides [24]. Initially, its inhibitory activity was tested using an agar diffusion assay [24] on 250 indicator strains, of which 240 strains (19 species) were Gram-positive. All the Gram-positive bacteria tested, with the exception of three out of 53 *S. aureus* strains, were sensitive (results not shown) tested. The MICs of garvicin KS against a representative selection of the strains are shown in Table 1. Garvicin KS inhibited pathogens like *Listeria monocytogenes, S. aureus*, and vegetative cells of *Bacillus cereus*. The effect of garvicin KS on spores were not tested. The most sensitive bacteria were strains of the lactic acid bacteria *Lactococcus* and *Enterococcus*, with MIC ranging from 5 to 80 BU/mL. 5 BU/mL corresponds to a mixture of 10 nM of each of the pure peptides of garvicin KS [24]. Concentrations to inhibit other Gram-positive strains varied from 320 to 2560 BU/mL.

**Table 1:** MIC_50_ values of garvicin KS against different strains

Bacterial strain	MIC (BU/mL)
*Acinetobacter baumannii* B1162	2560
*Acinetobacter calcoaceticus* B1165	2560
*Acinetobacter iwoffii* B1163	2560
*Bacillus cereus* LMGT 2805	640–1280
*Cellulomonas fimi* LMGT3232	10–20
*Escherichia coli* LMGT 3704	NI
*Enterococcus faecalis* LMGT 2333	40–80
*Enterococcus faecium* LMGT 2763	20–40
*Lactobacillus curvatus* LMGT 2353	80
*Lactobacillus plantarum* LMGT 2003	80
*Lactobacillus sakei* LMGT 2361	320
*Lactobacillus salivarius* LMGT 2787	320–640
*Lactococcus garvieae* LMGT 3390	80
*Lactococcus lactis* IL1403	5
*Lactococcus lactis* LMGT 2122^a^	5
*Leuconostoc gelidum* LMGT 2386	640–1280
*Listeria innocua* LMGT 2710	80
*Listeria ivanovii* LMGT 2813	320
*Listeria monocytogenes* LMGT 2604	160
*Pediococcus acidilactici* LMGT 2002	320
*Pediococcus pentosaceus* LMGT 2001	640
*Pseudomonas aeruginosa* LMGT 3294	NI
*Staphylococcus aureus* LMGT 3242	2560
*Streptococcus salivarius* LMGT 3597	320

*NI* no inhibition

^a^Nisin producer

Notably, garvicin KS was inhibitory to *A. baumannii, A. iwoffii*, and *A. calcoaceticus* (MIC = 2560 BU/mL). However, other Gram-negative bacteria, such as *E. coli* and *P. aeruginosa*, did not show any sensitivity to garvicin KS in this assay.

### Garvicin KS Acts Synergistically with Other Antimicrobials Against *S. aureus*

*Staphylococcus aureus* LMGT 3242 was the least sensitive Gram-positive bacterium tested in the microtiter assay, and in time-kill assays we were unable to fully prevent growth of this bacterium even with high concentration of garvicin KS. We therefore used this strain to investigate the effects of combining garvicin KS with other antimicrobials, including nisin, another bacteriocin with a wide inhibitory spectrum [1] and farnesol, shown to act as adjuvant to antimicrobials in *Staphylococci* [17]. Using the checkerboard assay, we found synergy between nisin and garvicin KS (FIC = 0.22) and between bacteriocins and farnesol, (both with FICs of 0.47), and all three compounds in combination (FIC = 0.33) (Table 2). The three components mixture resulted in the reduction of MIC by a factor of 8 for farnesol, and by a factor of 10 for garvicin KS and nisin, respectively.

**Table 2:** MIC values of garvicin KS, nisin, farnesol alone and in combinations, and FICs of combinations against *Staphylococcus aureus* LMGT 3242

MIC^a^			MIC (FIC) in mixture			
Garvicin KS	Nisin	Farnesol	Garvicin KS + nisin	Nisin + farnesol	Garvicin KS + farnesol	Garvicin KS + nisin + farnesol
2560	25	0.6	312/2.50 (0.22)	5.00/0.16 (0.47)	512/0.16 (0.47)	256/2.50/0.08 (0.33)

^a^Concentrations are given in BU/mL for garvicin KS, µg/mL for nisin and mM for farnesol

**Fig. 1:**
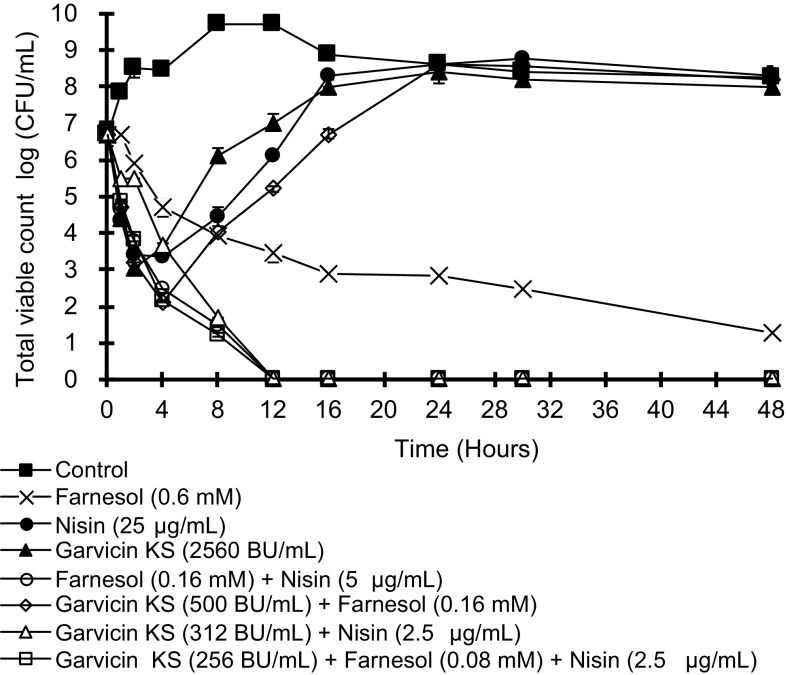
Time-killing analysis reveals the antimicrobial effects of garvicin KS, nisin and farnesol against *S. aureus* LMGT 3242. Viable counts were determined in triplicate

The effects of garvicin KS, nisin, and farnesol to *S. aureus* were also studied in time-kill assay. In the experiments, concentrations corresponding to the MIC (Table 2) were used. As shown in Fig. 1, none of the three antimicrobials alone could completely kill the bacteria even after 48 h exposure. When tested alone nisin and garvicin KS both caused an initial 3-log reduction in TVC before growth of survivors was detected. The mixtures nisin + garvicin KS caused a complete killing after about 12 h and no regrowth was seen even after 48 h, demonstrating the strong synergy between the two bacteriocins. Killing using the farnesol + garvicin KS mixture was less efficient, causing 4, 5 log reduction of CFU before the growth started. The farnesol + nisin mixture, on the other hand, gave complete killing after 12 h, as did the combination of all three compounds. We tested six other strains of *S. aureus*, including three MRSA, in the time-kill assay using the same concentrations as shown in Fig. 1. All strains were sensitive to nisin and garvicin KS, but none were eradicated by these bacteriocins alone or in combination. Only one of the six strains was completely killed by the mixture of nisin and farnesol. However, the mixture of farnesol, nisin, and garvicin KS caused complete killing of all six strains.

### Antimicrobial Synergy Against Gram-Negative Bacteria

A unique feature of garvicin KS is that it has antimicrobial effects on *Acinetobacter* spp., but relatively high amount of garvicin KS was required to inhibit them. Recently, farnesol was found to be a good adjuvant with polymyxin B to *A. baumannii* [18], and like for *S. aureus*; we tested farnesol in mixture with garvicin KS for synergy in inhibiting *Acinetobacter* spp.. No synergy was seen with these combinations (data not shown). We therefore replaced farnesol with polymyxin B in the mixtures.

The *A. baumannii* and *A. calcoaceticus* strains tested were both sensitive to polymyxin B with a MIC of 0.63 µg/mL, while *A. iwoffii* with a MIC of 25 µg/mL is considered resistant [2] (Table 3). Synergy was seen between polymyxin B and garvicin KS against all three *Acinetobacter* strains tested, even the polymyxin B resistant *A. iwoffii*. Similar synergistic effects were seen between nisin and polymyxin B. The combinations of three antimicrobial agents also showed synergistic effects against all the *Acinetobacter* strains. Compared to the individual antimicrobials combining the three compounds caused about 10-fold and 15-fold MIC reductions for garvicin KS and polymyxin B, respectively (Table 3).

**Table 3:** MIC values of garvicin KS, polymyxin B, and nisin alone and in combinations and FICs of combinations against *Acinetobacter* spp. and *Escherichia coli*

	MIC^a^			MIC (FIC) in mixture			
Bacterium	Garvicin KS	Nisin	Polymyxin B	Garvicin KS + nisin	Nisin + polymyxin B	Garvicin KS + polymyxin B	Garvicin KS + nisin + polymyxin B
*A. baumannii* B1162	2560	NI	0.63	2560/NI (2.0)	1.25/0.05 (0.08)	500/0.15 (0.44)	150/0.80/0.05 (0.20)
*A. iwoffii* B1163	2560	NI	25.0	2560/NI (2.0)	3.00/5.00 (0.20)	512/6.25 (0.45)	250/2.50/1.50 (0.16)
*A. calcoaceticus* B1165	2560	NI	0.63	2560/NI (2.0)	5.00/0.31 (0.50)	256/0.20 (0.42)	250/2.50/0.16 (0.35)
*E. coli* LMGT 3704	NI	NI	0.30	NI (X)	10.0/0.12 (0.40)	3200/0.12 (0.40)	3200/4.0/0.12 (0.40)

NI means no inhibition

X means not valid

^a^Concentrations are given in BU/mL for garvicin KS, µg/mL for nisin and polymyxin B

**Fig. 2:**
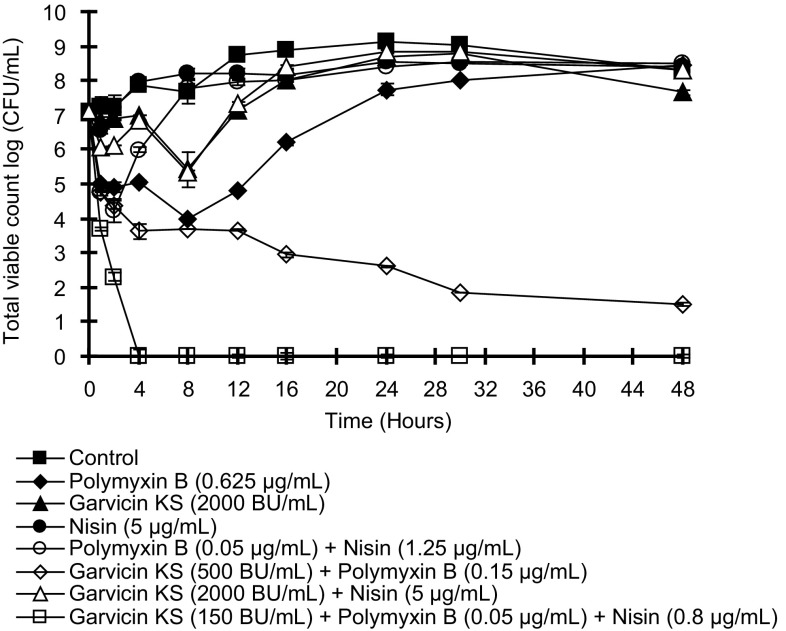
Time-killing analysis reveals the antimicrobial effects of garvicin KS, nisin and polymyxin B against *A. baumannii* B1162. Viable counts were determined in triplicate

The effects on *A. baumannii* of nisin, garvicin KS, polymyxin B alone and in mixtures were assayed in time-kill assays using concentrations corresponding to the MIC values shown in Table 3. As shown in Fig. 2, garvicin KS and polymyxin B could both kill the bacteria, but regrowth was seen after about 8 h and an initial 2–3 log killing in either case. Mixing garvicin KS and polymyxin B prevented regrowth of the bacteria, but the nisin + polymyxin B mixture did not. The bacteria were rapidly killed by the mixture of the three components, reaching eradication after 4 h.

**Fig. 3:**
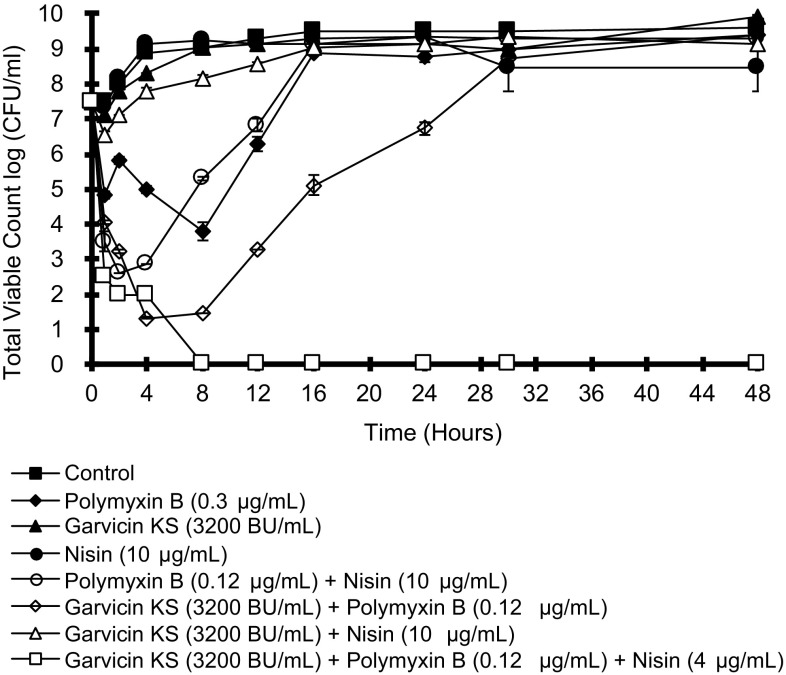
Time-killing analysis reveals the antimicrobial effects of garvicin KS, nisin, and polymyxin B against *E. coli* LMGT 3704. Viable counts were determined in triplicate

Prompted by the results using *Acinetobacter* strains, we extended the synergy studies to other Gram-negative bacteria. Garvicin KS and nisin were unable to kill *E. coli* alone, but the strain tested was sensitive to polymyxin B (MIC = 0.3 µg/mL). Both bacteriocins showed synergy with polymyxin B. A FIC of 0.4 was found for two- and three components mixture with polymyxin B (Table 3). The synergies were also demonstrated in time-kill assays **(**Fig. 3 **)**. Both bacteriocins improved the killing rates compared to polymyxin B alone, but regrowth was seen. The mixture of all three components, however, caused eradication of the bacteria after 8 h exposure.

We also tested synergy effects by using garvicin KS, nisin, and polymyxin B against *P. aeruginosa*. The MIC for polymyxin B against the strain tested was 0.8 µg/mL. However, there were no synergy effects on their combinations (data not shown).

## Discussion

Garvicin KS is a new bacteriocin comprised of three similar peptides of 32–34 amino acids [24]. The bacteriocin shows a broad inhibitory spectrum encompassing all the Gram-positive genera tested. Such a wide spectrum of activity is uncommon among bacteriocins, and has only been reported for certain lantibiotics like nisin and lactacin 3147 [11]. Of note, unlike the lantibiotics, garvicin KS was able to inhibit Gram-negative bacteria of *Acinetobacter* genus. However, the sensitivity towards garvicin KS varied more than 500-fold between the bacteria tested. Relatively high amounts of garvicin KS were required to kill many of the pathogenic species tested, and these bacteria were not completely killed and regrew after initial killing.

Combining antimicrobial agents offers a potential for increasing antimicrobial treatment efficacy and for reducing resistance evolution, and the use of combination antimicrobial therapy is widely used in the treatment of serious infections [4]. Importantly, the individual bacteriocins may act more effectively in combination with other antimicrobials agents [6], and studies demonstrating such effects have recently been reviewed [19]. Here, we have shown that garvicin KS is synergistic with other antimicrobial compounds, improving killing kinetics, and eradication and hence reducing resistance development. It is notable that garvicin KS could act synergistically with all the three compounds tested, indicating differences in mode of killing.

Importantly, garvicin KS showed synergy with polymyxin B, a drug of last resort in the combat infections by multidrug resistant Gram-negative bacteria but which is avoided due to its toxicity at relevant concentrations [9, 26]. Moreover, the emergence of resistance to polymyxins has been reported [8, 10]. Synergy between polymyxins and nisin and lacticin 3147, both bacteriocins from Gram-positive bacteria, against Gram-negative bacteria has been reported [13, 20, 21]. The synergy can be attributed to disruption of the outer membrane by polymyxin B allowing access of the bacteriocins to their target [7]. Polymyxin B is used for treatment of nosocomial infections mostly caused by *A. baumannii*, but because surviving bacteria are frequently found most patients are recommended to receive combination therapy of polymyxin B with other agents active against *A. baumannii* [23, 29]. The potential benefits of combining with new bacteriocins, like garvicin KS that act synergistically are obvious. Importantly, the concentration of polymyxin B needed to eradicate the bacteria was significantly reduced in combination with garvicin KS and nisin and much lower than the dosage recommended for polymyxin B therapy [29].

Strong synergy between nisin and garvicin KS was also observed against *S. aureus*. The synergy indicates that garvicin KS and nisin have different modes of action, which is different from classical antibiotics. Importantly, against *S. aureus* they were both synergistic with farnesol, a cheap and harmless compound which has been considered a promising adjuvant for antibiotics [15]. Moreover, the mixture of farnesol, nisin and garvicin KS completely killed all the *S. aureus* tested.

In conclusion, garvicin KS is a promising antimicrobial agent. We have demonstrated activity against a wide variety of bacteria, including pathogenic species known to account for a large number nosocomial infections, often with multi-resistant strains. The mode of action of garvicin KS is unknown, but different from many other used antimicrobials. Furthermore, garvicin KS mixtures with other antimicrobial compounds can be highly efficient by improving killing kinetics and eradication hence lower resistance development.
